# Discovery of genes required for lipoteichoic acid glycosylation predicts two distinct mechanisms for wall teichoic acid glycosylation

**DOI:** 10.1074/jbc.RA117.001614

**Published:** 2018-01-17

**Authors:** Jeanine Rismondo, Matthew G. Percy, Angelika Gründling

**Affiliations:** From the Section of Microbiology and Medical Research Council Centre for Molecular Bacteriology and Infection, Imperial College London, London SW7 2AZ, United Kingdom

**Keywords:** Gram-positive bacteria, Bacillus, Staphylococcus aureus (S. aureus), cell wall, glycosyltransferase, bacterial glycobiology, lipoteichoic acid, wall teichoic acid, teichoic acid, glycosylation

## Abstract

The bacterial cell wall is an important and highly complex structure that is essential for bacterial growth because it protects bacteria from cell lysis and environmental insults. A typical Gram-positive bacterial cell wall is composed of peptidoglycan and the secondary cell wall polymers, wall teichoic acid (WTA) and lipoteichoic acid (LTA). In many Gram-positive bacteria, LTA is a polyglycerol-phosphate chain that is decorated with d-alanine and sugar residues. However, the function of and proteins responsible for the glycosylation of LTA are either unknown or not well-characterized. Here, using bioinformatics, genetic, and NMR spectroscopy approaches, we found that the *Bacillus subtilis csbB* and *yfhO* genes are essential for LTA glycosylation. Interestingly, the *Listeria monocytogenes* gene *lmo1079*, which encodes a YfhO homolog, was not required for LTA glycosylation, but instead was essential for WTA glycosylation. LTA is polymerized on the outside of the cell and hence can only be glycosylated extracellularly. Based on the similarity of the genes coding for YfhO homologs that are required in *B. subtilis* for LTA glycosylation or in *L. monocytogenes* for WTA glycosylation, we hypothesize that WTA glycosylation might also occur extracellularly in *Listeria* species. Finally, we discovered that in *L. monocytogenes*, *lmo0626* (*gtlB*) was required for LTA glycosylation, indicating that the encoded protein has a function similar to that of YfhO, although the proteins are not homologous. Together, our results enable us to propose an updated model for LTA glycosylation and also indicate that glycosylation of WTA might occur through two different mechanisms in Gram-positive bacteria.

## Introduction

The bacterial cell wall is a highly complex and very important structure; it maintains the cell shape and protects bacteria from cell lysis and environmental insults. The main cell wall components present in Gram-positive bacteria, such as *Bacillus subtilis*, *Listeria monocytogenes*, and *Staphylococcus aureus*, are peptidoglycan and teichoic acids. Teichoic acids are anionic carbohydrate-containing polymers that are present in two forms: wall teichoic acid (WTA),^4^ which is covalently linked to the *N*-acetylmuramic acid residues of the peptidoglycan polymer, and lipoteichoic acid (LTA), which is embedded in the cytoplasmic membrane via a lipid anchor (1–3). Recent studies have shown that WTA is crucial for the virulence and β-lactam resistance of *S. aureus* and *L. monocytogenes* (4–6), whereas LTA is important for cell viability and cell division in these human pathogens (7, 8). In the soil bacterium *B. subtilis*, the absence of LTA affects divalent cation homeostasis and leads to increased sensitivity to diverse antibiotics and lysozyme, and the absence of WTA leads to drastic morphological alterations (9). It has also been shown that the deficiency in LTA synthesis leads to smaller colony sizes due to a failure in the execution of the colony developmental program in *B. subtilis* (10). Additionally, *B. subtilis* cells lacking both WTA and LTA are not viable, reflecting the importance of these cell polymers (9). Due to the impact of WTA and LTA on cell viability and virulence, the enzymes required for their synthesis are considered suitable targets for the development of new antimicrobial compounds (11–13).

The biosynthesis of LTA has been extensively studied in *B. subtilis*, *S. aureus*, and *L. monocytogenes* (3, 8, 14–17). *B. subtilis* produces type I LTA, which is composed of an unbranched 1–3-linked polyglycerol-phosphate (GroP) backbone chain that is attached to the outer layer of the bacterial membrane via a glycolipid anchor (14, 16). Each GroP subunit can be modified with d-alanine or GlcNAc residues (18–21). *L. monocytogenes* also produces a type I LTA; however, in this organism, the GroP subunits are substituted with d-alanine and galactose residues (22, 23). The enzymes required for the d-alanylation of LTA are encoded by the *dltABCD* operon and have been characterized in a number of studies (2, 24, 25). In contrast to the d-alanylation process, little is known about the enzymes responsible for LTA glycosylation. Fischer and others proposed a model for the addition of sugar residues to LTA based on biochemical studies performed three decades ago (21, 26–28). According to this model, a cytoplasmic glycosyltransferase (GT) uses a nucleotide-activated sugar to form a C_55_-P sugar intermediate, which is subsequently transported across the membrane by a flippase enzyme. The sugar molecule is subsequently transferred onto LTA by the action of a second GT (27, 28). As the polyglycerol-phosphate backbone of LTA is polymerized on the outside of the cell, this final step needs to be catalyzed by a GT with an extracellular active site. Recently, GtlA (locus tag Lmo0933 in strain EGD-e) has been identified as the putative cytoplasmic GT involved in the glycosylation process of LTA in the *L. monocytogenes* strain 10403S (29), although biochemical evidence for such an activity is still lacking. This protein is anchored by two C-terminal transmembrane helices to the membrane and contains a large N-terminal cytoplasmic enzymatic domain. NMR analysis of LTA produced by a *gtlA* mutant strain confirmed the absence of galactose modifications. Additionally, cell extracts obtained from the *gtlA* mutant strain showed a stronger LTA signal on western blots using a polyglycerol phosphate–specific antibody as compared with a WT strain, suggesting that the LTA backbone structure is better recognized by the antibody in the absence of sugar modifications (29). GtlA belongs to the GT2 family of glycosyltransferases and is characterized by a GT-A fold. GT-A fold glycosyltransferases assume a Rossmann fold with seven or more β-sheets, which is typical for proteins that bind nucleotides (30, 31); in *L. monocytogenes*, the likely substrate of GtlA is UDP-galactose. A second feature of enzymes with a GT-A fold is the presence of a conserved D*X*D motif, which interacts with the phosphate group of the nucleotide-activated sugar. This interaction requires a divalent cation, which in many cases is a Mn^2+^ ion (31–33). However, the remaining proteins required for the glycosylation of LTA in *L. monocytogenes* are still unknown. Also, none of the enzymes involved in the LTA glycosylation process of *B. subtilis*, including the enzyme required for the production of the C_55_-P sugar intermediate, the enzyme required for the transfer of this intermediate from the inner to the outer leaflet of the membrane, or the enzyme that transfers the sugar residue to the polyglycerol backbone, have been identified. Here, we show that deletion of the *csbB* and *yfhO* genes in *B. subtilis* led to a loss of sugar modifications on LTA. Interestingly, the *L. monocytogenes* YfhO homolog, Lmo1079, is not involved in LTA but WTA glycosylation. Instead, we found that the absence of Lmo0626 (here renamed GtlB) has an impact on LTA glycosylation, and we hypothesize that this protein performs the extracellular LTA glycosylation step in *L. monocytogenes.* With this, not only did we discover additional genes required for LTA glycosylation, but the work also allowed us to propose an alternative, extracellular, glycosylation mechanism for wall teichoic acid.

## Results

### CsbB is required for LTA glycosylation in B. subtilis

**Figure 1.**
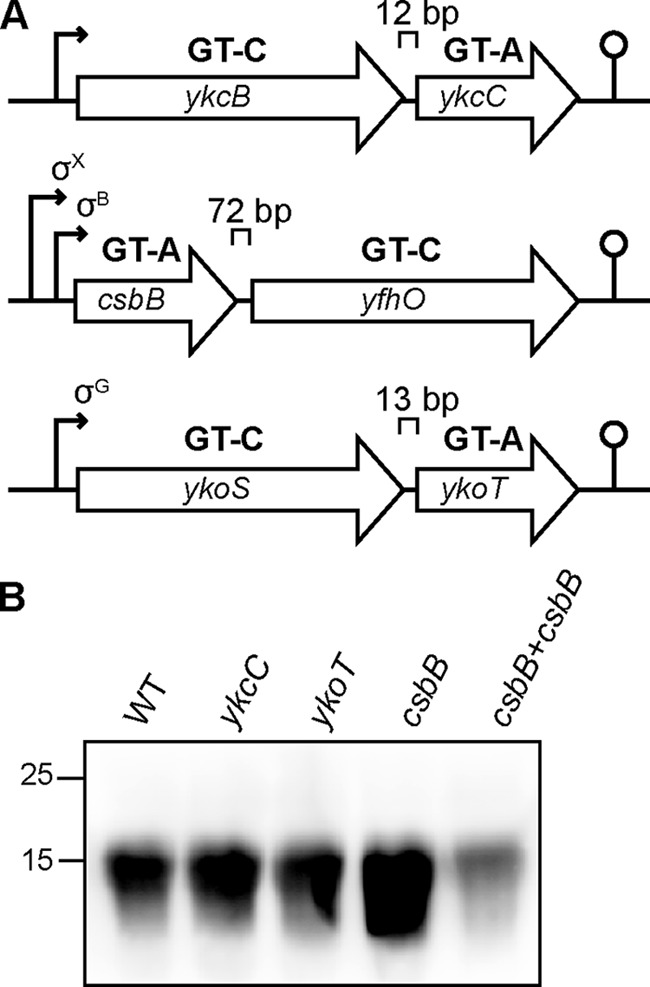
***B. subtilis* operons coding for *L. monocytogenes* GtlA homologs.** *A*, *B. subtilis* operons coding for predicted GT-A-fold glycosyltransferases YkcC, CsbB, and YkoT with homology to the *L. monocytogenes* GtlA enzyme and the predicted GT-C-fold glycosyltransferases YkcB, YfhO, and YkoS, respectively. The distance between adjacent genes is given in base pairs (*bp*), and the gene orientation is indicated by the *arrowheads*. Promoters are indicated by *black arrows*, and transcription terminators are indicated by a *loop structure*. The *ykcBC* operon is part of the YclJ and YrkP regulon (82, 83). Earlier studies indicated that the *csbB-yfhO* operon is expressed from σ^X^- and σ^B^-dependent promoters and that the *ykoST* operon is expressed from a σ^G^-dependent promoter (40, 48, 71, 84). *B*, LTA analysis by western blotting. Cell extracts of *B. subtilis* strains 168 (WT), *ykcC*, *ykoT*, *csbB* mutants, and the complementation strain *csbB*+*csbB* were prepared and separated on a 15% SDS-polyacrylamide gel. LTA was detected by western blotting using a humanized monoclonal LTA-specific antibody and an HRP-linked anti-human antibody. A representative blot from four independent experiments is shown.

**Figure 2.**
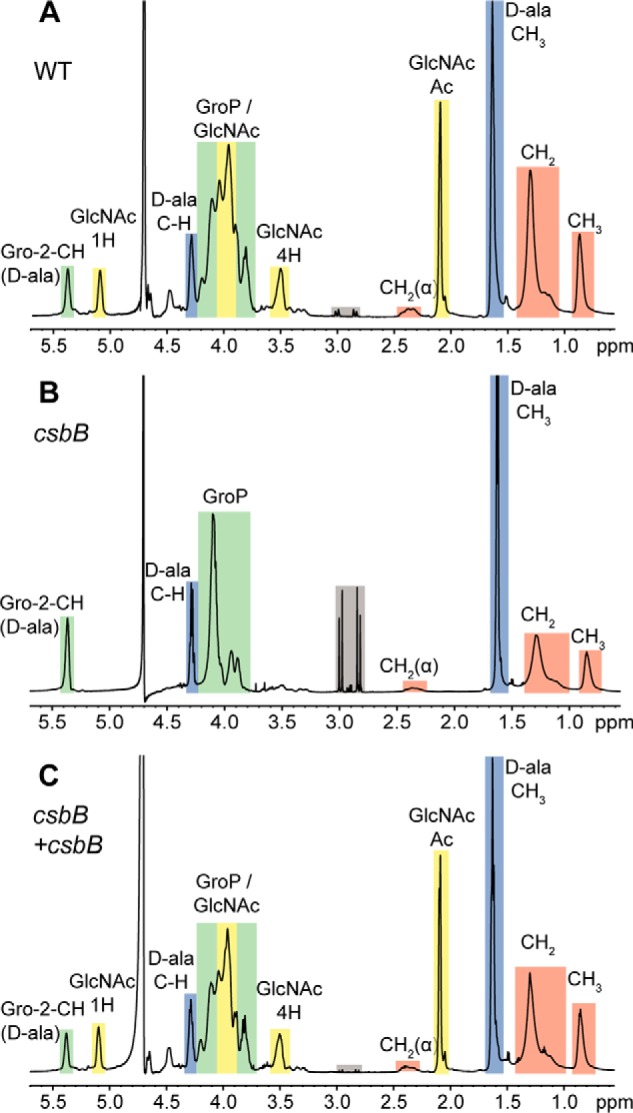
**NMR analysis of LTA isolated from WT *B. subtilis* 168, *csbB* mutant, and complementation strains.** Shown are NMR spectra of LTA derived from *B. subtilis* strains 168 (WT) (*A*), *csbB* mutant (*B*), and the *csbB*+*csbB* complementation strain (*C*). *Colored boxes* and *labels* indicate nonexchangeable protons derived from the different LTA components. Peaks were assigned as described previously (17, 37–39). The different peaks for the protons and acetyl group of GlcNAc are *labeled* with *1H*, *4H*, and *Ac*, respectively. *Gray boxes* highlight peaks resulting from residual citrate, a buffer component used during the LTA purification procedure. The spectra are representative of three independent experiments.

We have recently reported that the annotated glycosyltransferase GtlA probably catalyzes the first step of the LTA glycosylation process in *L. monocytogenes* (29). However, the enzymes involved in this process in other bacteria, including *B. subtilis*, remain unknown. To identify proteins required for LTA glycosylation in *B. subtilis*, the *L. monocytogenes* GtlA protein sequence was used as a query sequence in a BLASTP search against the *B. subtilis* 168 genome. This identified three homologs, YkcC (*e*-value: 1*e*−158), CsbB (*e*-value: 5*e*−68), and YkoT (*e*-value: 1*e*−60), all of which are encoded in a two-gene operon (Fig. 1*A*). Analysis using the Pfam database (http://pfam.xfam.org/)^5^ indicated that YkcC, CsbB, and YkoT encode GT-A fold family 2 glycosyltransferases, which are known to transfer sugar moieties from nucleotide-activated sugars, such as UDP-glucose, UDP-GlcNAc, or UDP-galactose to a variety of substrates, including the lipid carrier C_55_-P (34–36). To assess whether YkcC, CsbB, and YkoT are involved in the LTA glycosylation process in *B. subtilis*, *ykcC*, *csbB*, and *ykoT* deletion strains were constructed by replacing the respective gene in *B. subtilis* 168 with an antibiotic resistance marker. To determine whether deletion of one of these genes impacts LTA synthesis, anti-LTA western blot analysis was performed on cell extracts derived from the WT and mutant *B. subtilis* strains. The LTA isolated from strain 168Δ*csbB* yielded a stronger signal as compared with the WT strain (Fig. 1*B*), indicating that the structure or the amount of the LTA polymer is changed in the absence of CsbB. In contrast, the anti-LTA signal for strains with a *ykcC* or *ykoT* deletion was indistinguishable from that seen for the WT (Fig. 1*B*). To determine the chemical structure of LTA in the WT and mutant strains, the polymer was isolated and analyzed by 1D ^1^H NMR. LTA purified from WT *B. subtilis* 168 showed the expected spectrum; peaks derived from the CH_2_ groups of the GroP backbone (*colored green* in Fig. 2*A*), the CH_2_ and CH_3_ groups of the fatty acid chain (*colored orange* in Fig. 2*A*), the non-exchangeable protons from the d-Ala substitutions (*colored blue* in Fig. 2*A*), and the GlcNAc modification (*colored yellow* in Fig. 2*A*) could be assigned, as described previously (29, 37–39). The spectra, including the peaks for the nonexchangeable protons derived from the GlcNAc residues, were essentially identical for the LTA isolated from the *ykcC* and *ykoT* deletion strains to that of the WT strain, indicating that the proteins encoded by these two genes are not involved in the LTA glycosylation process in *B. subtilis* under the conditions used (Fig. S1). In contrast, the GlcNAc-specific peaks were absent in the NMR spectra obtained from the LTA isolated from the *csbB* mutant (Fig. 2*B*). To confirm that the phenotype is due to inactivation of *csbB*, a copy of *csbB* expressed from its native promoter was introduced into the *amyE* locus of the *csbB* mutant. The LTA western blot signal (Fig. 1*B*) and NMR peaks corresponding to GlcNAc (Fig. 2*C*) were restored to WT levels in the complementation strain. These results highlight that CsbB is required for the decoration of LTA with GlcNAc residues in *B. subtilis* during vegetative growth. Although biochemical evidence is still lacking, we presume that CsbB functions as the cytoplasmic LTA glycosyltransferase.

### The B. subtilis YfhO protein is involved in the LTA glycosylation process in B. subtilis

**Figure 3.**
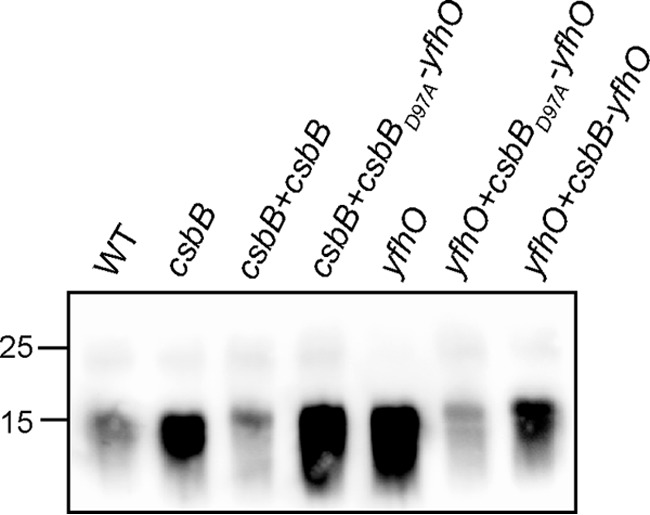
**LTA production in WT *B. subtilis* 168, *csbB*, and *yfhO* deletion and complementation strains.** Cell extracts were prepared from overnight cultures of *B. subtilis* 168 (WT), *csbB*, *csbB*+*csbB*, *csbB*+*csbB_D97A_-yfhO*, *yfhO*, *yfhO*+*csbB_D97A_-yfhO*, and *yfhO+csbB-yfhO* mutant and complementation strains and separated on a 15% SDS-polyacrylamide gel. The LTA was detected by western blotting using a humanized monoclonal LTA-specific antibody and an HRP-linked anti-human antibody. A representative blot from five independent experiments is shown.

**Figure 4.**
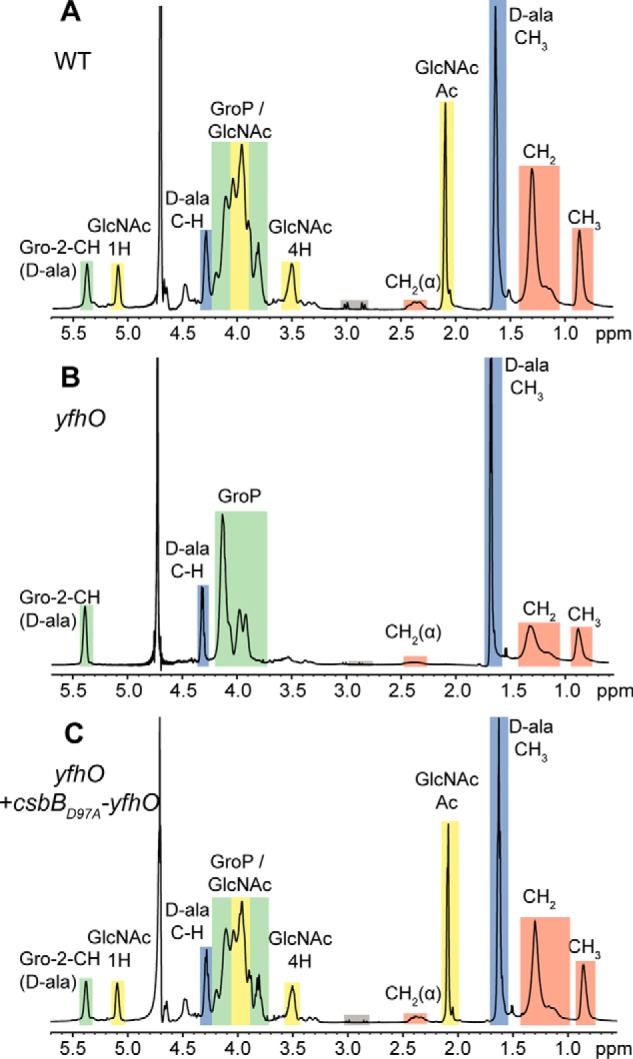
**NMR analysis of LTA isolated from *B. subtilis* wildtype 168, *yfhO* mutant, and complementation strains.** *A–C*, NMR spectra of LTA obtained from *B. subtilis* 168 (WT) (*A*), the *yfhO* mutant (*B*), and the *yfhO*+*csbB_D97A_-yfhO* complementation strain (*C*). *Colored boxes* and *labels* indicate nonexchangeable protons derived from the different LTA components. Peaks were assigned as described previously (17, 37–39). The different peaks for the protons and acetyl group of GlcNAc are *labeled* with *1H*, *4H*, and *Ac*, respectively. *Gray boxes* highlight peaks resulting from residual citrate, a buffer component used during the LTA purification procedure. The spectra are representative of three independent experiments. The spectrum for the *B. subtilis* 168 (WT) LTA is the same as shown in Fig. 2*A*.

Analysis of the genomic *ykcC*, *csbB*, and *ykoT* regions highlighted that all three genes are encoded in an operon with a second gene, namely *ykcB*, *yfhO*, and *ykoS*, respectively (Fig. 1*A*). YkcB, YfhO, and YkoS show similarities to members of the GT-C family glycosyltransferases, which are characterized by 8–13 transmembrane helices and a D*X*D or modified (D*X*E, E*X*D, DD*X*, or DE*X*) motif in an extracellular loop (34). GT-C enzymes are good candidate enzymes for transferring the glycosyl group from the lipid intermediate to the LTA backbone on the outside of the cell. Because CsbB is required for LTA glycosylation in *B. subtilis*, we hypothesized that YfhO, the predicted GT-C enzyme encoded in the same operon, could also be involved in the LTA glycosylation process. To test this, a *yfhO* mutant was constructed, and the LTA isolated from this strain was analyzed by western blotting. Similar to the *csbB* mutant, the LTA signal for the *yfhO* deletion strain was stronger than that of the WT strain (Fig. 3). This phenotype could be complemented by expressing the complete *csbB-yfhO* operon from the native promoter from the *amyE* locus in the *yfhO* mutant strain (*yfhO+csbB-yfhO*; Fig. 3). To ensure that the complementation does not result from the overexpression of *csbB*, the *csbB_D97A_-yfhO* operon, which produces a non-functional CsbB variant, was constructed and introduced into the *yfhO* mutant and the *csbB* mutant. Previous work had shown that the CsbB_D97A_ variant, in which the second Asp residue of the conserved D*X*D motif predicted to be required for binding of a divalent cation is mutated, is inactive (40). An increased LTA signal was observed on western blots for cell extracts obtained from the *B. subtilis csbB*+*csbB_D97A_-yfhO* strain, and no GlcNAc-specific peaks were observed by NMR analysis of purified LTA, confirming the inactivity of CsbB_D97A_ (Fig. 3 and Fig. S2). On the other hand, introduction of the *csbB_D97A_-yfhO* operon into the *yfhO* background strain led to partial complementation of the phenotype (Fig. 3). These data indicate that YfhO is involved in the LTA glycosylation process in *B. subtilis*. To investigate this further, LTA was isolated from the *yfhO* mutant and the *yfhO*+*csbB_D97A_-yfhO* complementation strain, as well as the WT control strain, and analyzed by NMR. GlcNAc-specific peaks were absent in the sample derived from the *yfhO* deletion strain, but again present in the complementation strain (Fig. 4). We also constructed *B. subtilis ykcB* and *ykoS* mutants, with deletions in the other two genes coding for predicted GT-C–like glycosyltransferases. However, LTA glycosylation was not abolished in these strains under the conditions tested (Fig. S3). Taken together, these data show that YfhO is involved in the LTA glycosylation process in *B. subtilis.* Although biochemical evidence is still lacking, we hypothesize that the *B. subtilis* YfhO protein probably acts as extracellular GT-C-type glycosyltransferase and moves the GlcNAc sugar moieties from a C_55_-P intermediate onto the LTA backbone on the outside of the cell.

### LTA glycosylation has no impact on chain length and percentage of d-alanine substitutions in B. subtilis

**Figure 5.**
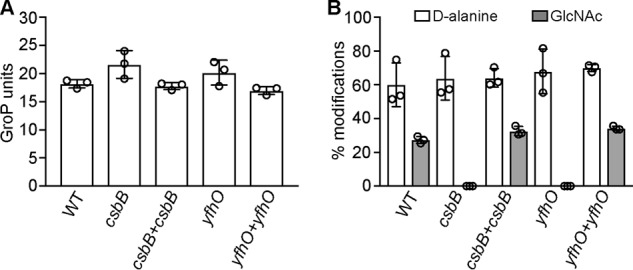
**Determination of LTA chain length and percentages of d-alanine and GlcNAc substitutions.** The peaks in the NMR spectra obtained from LTA isolated from *B. subtilis* strains 168 (WT), *csbB*, *csbB+csbB*, *yfhO*, and *yfhO+csbB_D97A_-yfhO* (*yfhO+yfhO*) were integrated, and the chain length (*A*) and percentages of d-alanine and GlcNAc substitutions (*B*) were determined. The average values and S.D. (*error bars*) from three independent experiments were determined and plotted. Two-tailed unpaired *t* tests identified only significant differences for the percentage of GlcNAc substitution between the WT *B. subtilis* 168 and *csbB* or *yfhO* mutant strains with *p* values < 0.05.

The polymerization of the LTA backbone takes place on the outside of the cell, and hence the transfer of d-alanine and sugar residues must also take place on the outside of the cell. Furthermore, individual glycerol-phosphate subunits are modified with either a d-alanine or sugar residue but not with both. It is conceivable that the LTA polymerization and modification processes are coordinated and that the absence of sugar modifications could impact the amount of d-alanine substitutions or even the chain length of LTA, in case sugar residues are introduced while the LTA backbone is still polymerized. To determine whether the chain length and/or the percentage of d-alanine modifications are changed in the absence of sugar modifications, the peaks of the NMR spectra were integrated, and the LTA chain length and percentage modification were calculated using a previously published method and as described under “Experimental procedures” (29). For each strain, three NMR spectra obtained from three independent LTA extractions were analyzed. The LTA isolated from WT *B. subtilis* 168 had a calculated chain length of 17.74 ± 1.04 GroP units, and 62.07 ± 11.32 and 27.05 ± 1.79% of the GroP residues were modified with d-alanine and GlcNAc residues, respectively (Fig. 5). No significant difference in chain length (21.62 ± 2.46 and 20.16 ± 2.19 GroP units; Fig. 5*A*) or percentage of d-alanine substitutions (63.89 ± 12.92 and 67.95 ± 13.15%; Fig. 5*B*) was observed for the *csbB* or *yfhO* mutant strain. The percentage of GlcNAc residues in the *csbB+csbB* and *yfhO+csbB_D97A_-yfhO* complementation strains were 33.74 ± 3.21 and 34.33 ± 1.22% and therefore comparable with the percentage of sugar substitutions present in the LTA isolated from WT *B. subtilis* strain 168 (Fig. 5*B*). Taken together, these data suggest that the LTA chain length and the amount of d-alanine substitutions are not affected by the presence or absence of sugar modifications in *B. subtilis* 168 when grown under standard laboratory conditions.

### The L. monocytogenes YfhO homolog Lmo1079 is involved in WTA but not LTA glycosylation

**Figure 6.**
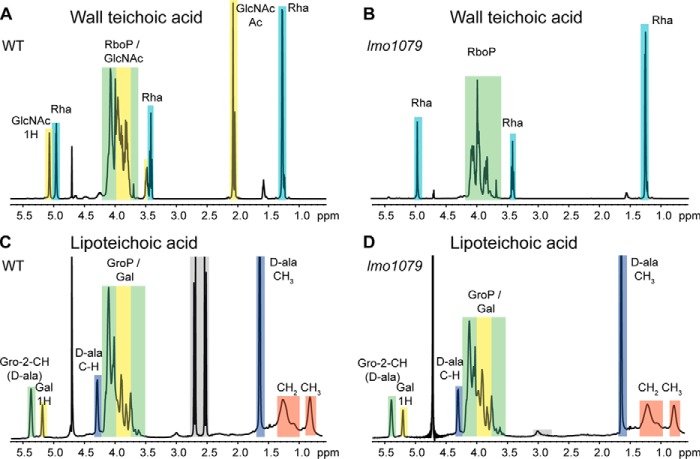
**Detection of sugar modifications on WTA and LTA using ^1^H NMR.** *A* and *B*, NMR spectra of WTA derived from *L. monocytogenes* 10403S (WT) (*A*) and the *lmo1079* mutant (*B*). Peaks of nonexchangeable protons were assigned to the different WTA components according to previously published spectra (45, 74, 78). The different peaks for the protons and acetyl group of GlcNAc are labeled with *1H*, *4H*, and *Ac*, respectively. One representative spectrum from three independent experiments is shown. *C* and *D*, NMR spectra of LTA derived from *L. monocytogenes* 10403S (WT) (*C*) and the *lmo1079* mutant strain (*D*). Peaks of nonexchangeable protons were assigned to the different LTA components using previously published spectra and are *highlighted* in *colored boxes* (29, 37–39). *Gray boxes* indicate residual citrate, a component of the buffer used for LTA purification. The spectra for *lmo1079* are representative of three independent experiments. LTA of strain 10403S was isolated once as a control.

So far, the only protein known to be involved in the LTA glycosylation process in *L. monocytogenes* is GtlA, an annotated GT with a predicted cytoplasmic catalytic domain (29). To identify additional *L. monocytogenes* proteins involved in the LTA glycosylation process, we performed a homology search using the *B. subtilis* YfhO protein as a query sequence. This yielded Lmo1079 as the only close homolog with an *e*-value of 1*e*−39. In previous work, it has been shown that deletion of *lmo1079* prevents binding of the phage-derived protein CBDP35, which recognizes terminal GlcNAc residues on the bacterial surface, to *L. monocytogenes* EGD-e cells (41). LTA in *L. monocytogenes* is modified with galactose residues, whereas terminal GlcNAc moieties are found on WTA in *L. monocytogenes* strains EGD-e and 10403S. Therefore, these previous findings indicate that Lmo1079 is probably required for the glycosylation of WTA with GlcNAc residues (41), but the structure of WTA produced by an *lmo1079* deletion strain has not yet been analyzed. To specifically determine the role of Lmo1079 in WTA glycosylation, and potentially in the glycosylation of LTA, a strain lacking *lmo1079* (10403SΔ*lmo1079*) was constructed. Next, the WTA and LTA polymers were isolated from WT 10403S and the *lmo1079* mutant and analyzed by NMR. As expected, GlcNAc moieties were present on the WTA polymer isolated from the WT strain but absent from the *lmo1079* mutant (Fig. 6, *A* and *B*). In contrast, no differences between the NMR spectra for LTA isolated from the WT and *lmo1079* mutant strains were observed, and galactose residues were present on the LTA isolated from both strains (Fig. 6, *C* and *D*). Taken together, these data show that Lmo1079 is involved in a different process than YfhO, its closest homolog in *B. subtilis*; Lmo1079 is needed for the glycosylation of WTA in *L. monocytogenes*, whereas YfhO is essential for LTA glycosylation in *B. subtilis*. The wider implications that closely related proteins are involved in WTA glycosylation in one bacterial species but LTA glycosylation in another bacterial species will be addressed in detail under “Discussion.”

### The L. monocytogenes GtlB protein is required for LTA glycosylation

**Figure 7.**
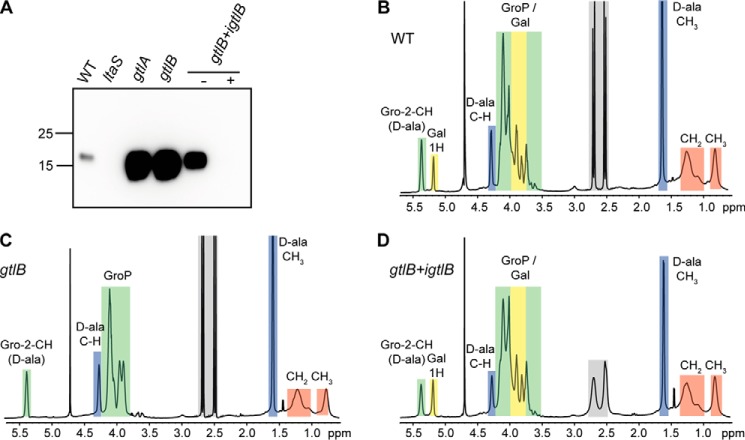
**LTA production in WT *L. monocytogenes* 10403S, the *gtlB* mutant, and the complementation strain.** *A*, LTA detection by western blotting. Cell extracts were prepared from the *L. monocytogenes* strains 10403S (WT), the *ltaS* and *gtlA* mutant control strains, and the *gtlB* mutant and complementation strain *gtlB+igtlB* grown in the absence (−) or presence (+) of IPTG. The extracts were separated on a 15% SDS-polyacrylamide gel, and the LTA was detected by western blotting using a polyglycerol phosphate–specific monoclonal antibody. Strains *ltaS* and *gtlA* were included as negative and positive control, respectively. *B–D*, NMR spectra of LTA isolated from strains 10403S (WT) (*B*), *gtlB* (*C*), and *gtlB+igtlB* (*D*) (grown in the presence of IPTG). *Colored boxes* and *labels* indicate nonexchangeable protons derived from the different LTA components. Peaks were assigned as described previously (29, 37–39). *Gray boxes* indicate residues of citrate, a buffer component used for the LTA purification. The spectra are representative of three independent experiments.

**Figure 8.**
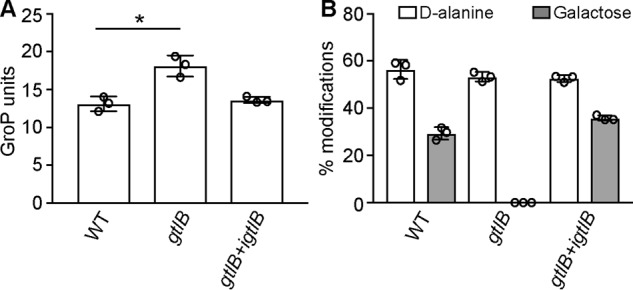
**Determination of chain length, percentage of d-alanine, and galactose modifications of LTA from WT *L. monocytogenes*, the *gtlB* mutant, and the complementation strain.** The different peaks in the NMR spectra obtained from LTA isolated from *L. monocytogenes* strains 10403S (WT), *gtlB*, and *gtlB+igtlB* were integrated, and the chain length (*A*) and percentages of d-alanine and galactose substitutions (*B*) were determined. The average values and S.D. (*error bars*) from three independent experiments were calculated and plotted. Two-tailed unpaired *t* tests identified significant differences for the number of GroP units of LTA isolated from the WT *versus gtlB* deletion strain with a *p* value < 0.05 (indicated by an *asterisk*).

Lmo1079 is the only *L. monocytogenes* protein that shows clear homology to the *B. subtilis* YfhO protein. However, as shown above, the protein could be excluded as the potential extracellular LTA GT in *L. monocytogenes*. Therefore, we used a bioinformatics approach to try to identify candidate proteins that could potentially act as extracellular GT involved in the LTA glycosylation process in *L. monocytogenes*. We reasoned that such a protein probably assumes a GT-C fold. As enzymes with such a fold have 8–13 transmembrane helices and a big extracellular loop containing the catalytic site of the protein (34), we performed a bioinformatics analysis to identify membrane proteins of unknown function in *L. monocytogenes* containing eight or more transmembrane helices and at least one extracellular loop of at least 50 amino acids. Genes coding for possible candidates were deleted in *L. monocytogenes* 10403S, and the LTA in these mutant strains was analyzed by western blotting. A strain with a deletion of *lmo0626*, which encodes a membrane protein with eight transmembrane helices and an extracellular loop of 61 amino acids, showed a similar increase in the anti-LTA signal as seen for the *gtlA* mutant (Fig. 7*A*). The *L. monocytogenes* protein Lmo0626 was renamed GtlB for glycosyltransferase LTA B. To confirm that the increased LTA signal is due to the deletion of *gtlB*, a complementation strain was constructed by introducing a functional copy of *gtlB* placed under IPTG-inducible expression control into the *gtlB* mutant strain, yielding strain 10403SΔ*gtlB* pIMK3*-gtlB* (*gtlB*+*igtlB*). Anti-LTA western blot analysis using cell extracts isolated from this strain grown in the absence of IPTG showed the expected increased LTA signal (Fig. 7*A*). However, the signal was not as strong as for extracts prepared from the original *gtlB* mutant, indicating low-level *gtlB* expression even in the absence of IPTG. When the *gtlB* complementation strain was grown in the presence of IPTG, the LTA signal was reduced, indicating successful complementation (Fig. 7*A*). Indeed, no signal was detected in the complementation strain, probably due to overexpression of GtlB upon IPTG induction (Fig. 7*A*). Next, LTA was isolated from WT 10403S, the *gtlB* mutant, and the complementation strain (*gtlB+igtlB*) grown in the presence of IPTG and subsequently analyzed by ^1^H NMR. The NMR spectra for the LTA isolated from the WT 10403S strain showed the expected peaks, which were assigned to the different LTA components according to published spectra (29, 37–39) (Fig. 7*B*). On the other hand, the peaks derived from the galactose residues were absent in the *gtlB* deletion strain but again present in the complementation strain (Fig. 7, *C* and *D*). To determine whether the absence of GtlB affects the LTA chain length and percentage of d-alanine substitutions, these parameters were calculated from the integral values of the different peaks. LTA isolated from WT 10403S had a chain length of 13.11 ± 0.95 GroP units, and 56.38 ± 4.05 and 29.32 ± 2.65% were modified with d-alanine and galactose, respectively (Fig. 8). Similar values were calculated for the LTA isolated from the *gtlB* complementation strain (13.62 ± 0.41 GroP units, 52.56 ± 1.37% d-alanine and 35.84 ± 1.08% galactose substitutions). Interestingly, deletion of *gtlB* did not only result in an absence of galactose residues, but the LTA polymer was also significantly longer with a calculated length of 18.11 ± 1.39 GroP units (Fig. 8*A*). On the other hand, the absence of *gtlB* did not influence the percentage of d-alanine modifications, which remained at 53.3 ± 1.99% (Fig. 8*B*). Altogether, these results show that GtlB is necessary for the glycosylation of LTA in *L. monocytogenes*, and we hypothesize that this protein functions as extracellular glycosyltransferase, similar to the *B. subtilis* YfhO protein, and transfers galactose residues onto the LTA backbone.

### Global analysis of GtlB proteins and identification of conserved aspartic acid residues required for function

**Figure 9.**
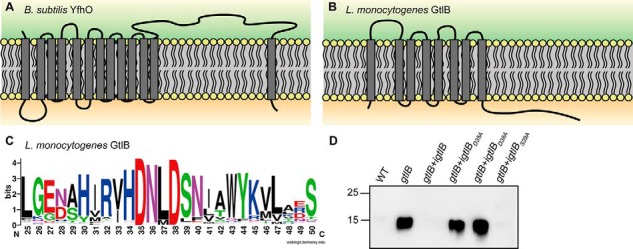
**Topology of YfhO and GtlB-like proteins and identification of conserved residues required for GtlB function.** *A* and *B*, topology models of *B. subtilis* YfhO (*A*) and *L. monocytogenes* GtlB (*B*) as predicted using the TMHMM version 2.0 server (85). *C*, WebLogo showing the conserved D*XX*D motif in the first extracellular loop of GtlB. Protein sequences of GtlB homologs with ≥20% identity were aligned in Jalview (80), and the alignment was used to create a WebLogo motif (81). The WebLogo motif for amino acid region 25–50 of GtlB is shown, with amino acids 35 and 38 being the conserved aspartic acid residues. *D*, LTA production in strains expressing different GtlB variants. *L. monocytogenes* strains 10403S (WT), *gtlB*, *gtlB+igtlB*, *gtlB+igtlB_D35A_*, *gtlB+igtlB_D38A_*, and *gtlB+igtlB_S39A_* were grown overnight in BHI medium at 37 °C in the presence of 1 mm IPTG. Cell extracts were prepared and separated on a 15% SDS-PAGE, and the LTA was detected using a polyglycerol phosphate–specific monoclonal LTA antibody. A representative image of three independent experiments is shown.

Although the *L. monocytogenes* GtlB and *B. subtilis* YfhO proteins show only limited sequence identity, our results suggest that both proteins are involved in the LTA glycosylation process. The *B. subtilis* YfhO protein forms part of the YfhO-domain Pfam protein family PF09586. Currently, more than 1800 sequences in over 1000 species are listed in this Pfam family with a large number of proteins found in Firmicutes, but also some in Actinobacteria and Bacteroidetes. Consistent with our hypothesis that YfhO acts as a glycosyltransferase, some members of this family are annotated GT-C glycosyltransferases, which are characterized by 8–13 transmembrane helices and a D*X*D or modified D*X*E, E*X*D, DD*X*, or DE*X* motif, followed by a short stretch of hydrophobic residues (34, 42). *B. subtilis* YfhO has 13 predicted transmembrane helices and a large extracellular loop of 389 amino acids connecting the last two transmembrane helices (Fig. 9*A*). Two possible D*X*D motifs, D^450^LD and D^480^DD, are present in this loop of the *B. subtilis* YfhO protein. However, a Jalview alignment of 870 *B. subtilis* YfhO homologs, which share at least 30% sequence identity, indicated that neither of these D*X*D motifs is conserved, making the prediction of residues potentially involved in enzyme catalysis difficult. No Pfam family is currently available for proteins that are homologous to the *L. monocytogenes* GtlB protein. Using the *L. monocytogenes* GtlB protein sequence as a query sequence in a BLASTP search, just over 1000 proteins with >20% identity could be identified. These proteins are mainly present in *Listeria* and *Bacillus* sp. (Table S3). The *L. monocytogenes* GtlB protein has only eight predicted transmembrane helices and a much smaller extracellular loop, which connects the first and second transmembrane helices (Fig. 9*B*). Although no D*X*D or modified D*X*D motif could be identified, GtlB-like proteins contain a conserved D*XX*D (D^35^NLD^38^ in GtlB) motif in this loop (Fig. 9*C*), and these aspartic acid residues could potentially form part of an active site. In addition, several highly conserved hydrophobic residues are found downstream of the D*XX*D motif, which is similar to what has been reported for the active sites of other GT-C-fold glycosyltransferases (Fig. 9*C*). To test whether the conserved aspartic acid residues are essential for the function of GtlB, an alanine mutagenesis experiment was performed. *gtlB* alleles with the appropriate base substitutions to change amino acids Asp^35^, Asp^38^, and as control Ser^39^ to an alanine were placed under IPTG-inducible expression control and integrated into the chromosome of the *L. monocytogenes gtlB* mutant strain, allowing for IPTG-dependent expression of the different GtlB variants. Cell extracts of strains expressing *gtlB*, *gtlB_D35A_*, *gtlB_D38A_*, and *gtlB_S39A_* were prepared, and the LTA was detected by western blot (Fig. 9*D*). As described earlier, an increased signal was observed for a *gtlB* mutant containing the empty plasmid vector, and the signal was again reduced upon expression of a functional GtlB protein (Fig. 9*D*). The expression of the GtlB_S39A_ variant complemented the *gtlB* mutant phenotype, as indicated by the weak anti-LTA signal, suggesting that residue Ser^39^ is dispensable for GtlB function (Fig. 9*D*). However, no complementation was observed in strains expressing the GtlB_D35A_ and GtlB_D38A_ variants (Fig. 9*D*). These data highlight that the aspartate residues at positions 35 and 38 in GtlB, with a predicted location in the first extracellular loop, are required for protein function and hence could form part of an extracellular active site. The *L. monocytogenes* strain used in this study is a 1/2a serovar strain; however, based on flagella and cell wall (somatic) antigens, 13 different *L. monocytogenes* serovars have been described (43, 44). Differences in WTA structure and glycosylation pattern provide the bases for differences in the somatic antigen (45), and for instance GlcNAc modifications are not present on WTA in all *L. monocytogenes* serovars (46, 47). Consistent with this, close homologs to Lmo1079, which is required in *Listeria* for the addition of GlcNAc residues onto WTA are only present in some but not all *L. monocytogenes* serovars (Table S3). On the other hand, a close homolog to GtlB, which is required for LTA glycosylation, is present in all 13 *Listeria* serovars as well as *Listeria innocua*, *Listeria ivanovii*, and *Listeria seeligeri* (Table S3), indicating that the LTA is probably modified with galactose residues in these strains.

## Discussion

As part of this work, we identified several new proteins required for the glycosylation of LTA in *B. subtilis* and *L. monocytogenes* and, as described below, propose a new model for the WTA glycosylation process in *L. monocytogenes.* In previous work, GtlA (Lmo0933) was shown to be involved in the glycosylation of LTA in *L. monocytogenes* (29). The three close *B. subtilis* homologs, YkcC, YkoT, and CsbB, are predicted glycosyltransferases with N-terminal cytoplasmic catalytic domains that assume a GT-A fold and are linked by two C-terminal transmembrane helices to the membrane. Here, we show that a *B. subtilis csbB* mutant, but not mutants with deletions in the other two genes, lacks GlcNAc modifications on LTA (Figs. 1*B* and 2*B* and Fig. S1). Previously, it was speculated that CsbB functions in a cell wall synthesis–related process (48, 49), and our study shows that this process is the glycosylation of the cell wall polymer LTA.

**Figure 10.**
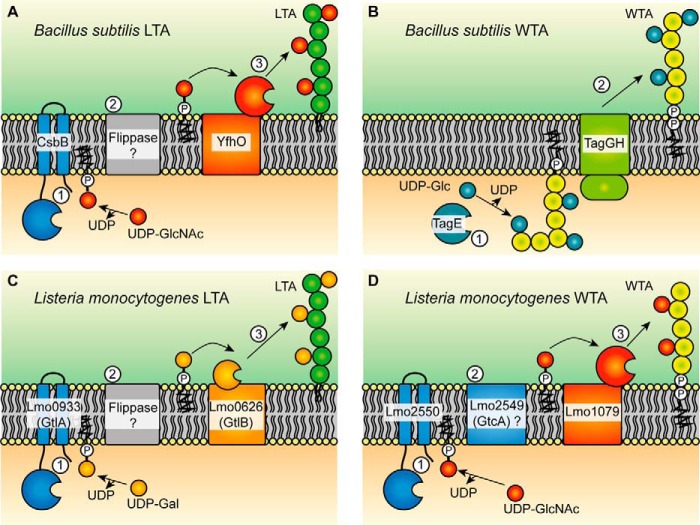
**Models for the LTA and WTA glycosylation processes in *B. subtilis* and *L. monocytogenes*.** *A* and *B*, proposed model for the glycosylation process of LTA (*A*) and WTA (*B*) in *B. subtilis. A*, based on the genetic data presented in this study, we suggest that LTA in *B. subtilis* is glycosylated with the aid of the cytoplasmic GT CsbB, which we predict transfers GlcNAc residues to the C_55_-P lipid carrier (*step 1*). The C_55_-P-GlcNAc intermediate is then transported across the membrane by an unknown flippase enzyme (*step 2*). We further hypothesize that the GlcNAc residues are then transferred onto LTA by the GT candidate enzyme YfhO (*step 3*). *B*, WTA of *B. subtilis* is modified with glucose residues in a process that takes place in the cytoplasm of the cell and that is catalyzed as previously reported by the GT TagE, which uses UDP-glucose as substrate (*step 1*) (58). The glycosylated WTA is subsequently transported across the membrane via TagGH (*step 2*) (57). *C* and *D*, proposed model for the LTA (*C*) and WTA (*D*) glycosylation process in *L. monocytogenes. C*, LTA in *L. monocytogenes* is modified with galactose residues. In the first step, GtlA, which is thought to act as cytoplasmic GT, transfers galactose molecules onto the C_55_-P lipid carrier (29). The sugar-lipid intermediate is then transported across the membrane by an unknown flippase enzyme (*step 2*), and the galactose residues are subsequently transferred onto LTA on the outside of the cell, and we suggest that this step is catalyzed by GtlB (*step 3*). *D*, for WTA glycosylation in *L. monocytogenes*, the cytoplasmic GT Lmo2550 probably produces a C_55_-P-GlcNAc lipid intermediate (*step 1*) that is subsequently transported across the membrane by a flippase, a function that might be provided by Lmo2549 (also referred to as GtcA) (*step 2*). As final step, we suggest that Lmo1079, a *B. subtilis* YfhO homolog, transfers the GlcNAc molecule onto WTA on the outside of the cell (*step 3*).

Fischer and others presented a model for the LTA glycosylation process in which a cytoplasmic GT initially transfers a sugar moiety onto a C_55_-P lipid carrier (21, 26–28). After the carrier is “flipped” from the inner leaflet to the outer leaflet of the membrane by a flippase enzyme, a second extracellular GT was suggested to transfer the sugar moiety onto the GroP units of LTA (21, 26–28). Due to the similarity of CsbB to the C_55_-P glycosyltransferase GtrB in Gram-negative bacteria, Inoue *et al.* (40) suggested that CsbB can glycosylate the lipid carrier C_55_-P. This is in good agreement with our hypothesis that CsbB produces a C_55_-P–GlcNAc intermediate (Fig. 10*A*). *csbB* is co-transcribed with *yfhO* (40), and the data presented in our study show that its absence leads to the lack of sugar modifications on LTA, suggesting that *yfhO* might encode a GT-C-fold glycosyltransferase responsible for the transfer of the GlcNAc residues onto the LTA polymer on the outside of the cell (Figs. 4*B* and 10*A*). YfhO is a membrane protein with 13 predicted transmembrane helices and a large extracellular loop between the last two transmembrane helices (Fig. 9*A*), which could aid in the transfer of the GlcNAc residues onto the LTA chain (Figs. 4*B* and 10*A*). The identity of the protein that facilitates the flipping of the C_55_-P–GlcNAc intermediate across the membrane remains unknown; potentially, YfhO could be responsible for the flipping of the C_55_-P–sugar intermediate in addition to the transfer of the sugar onto LTA, which will be interesting to experimentally test in future biochemical studies.

Not only LTA, but also WTA, is glycosylated in Gram-positive bacteria. This process is best characterized in *S. aureus*, where two cytoplasmic GTs, TarM and TarS, transfer α- and β-GlcNAc residues, respectively, onto the growing WTA chain (4, 50–52). Once glycosylated, the WTA polymer is transported across the bacterial membrane by the TarGH ABC transporter and subsequently linked to peptidoglycan by Lcp enzymes (53–56). WTA of *B. subtilis* is modified with glucose residues by the glycosyltransferase TagE in the cytoplasm of the cell and subsequently moved across the membrane by the TagGH transporter (Fig. 10*B*) (57, 58). Glycosylation of WTA can take place in the cytoplasm of the cell as WTA is polymerized within the cell. This is in contrast to polyglycerol-phosphate LTA, which is polymerized on the outside of the cell and hence can only by glycosylated on the extracellular side of the membrane. However, based on the homology of the *L. monocytogenes* WTA glycosylation enzymes with the enzymes involved in the *B. subtilis* LTA glycosylation process uncovered as part of this work, we propose that glycosylation of WTA might not universally take place in the cytoplasm of the cell. Instead, we hypothesize that the glycosylation of WTA in *L. monocytogenes* 10403S and EGD-e, 1/2a serovar strains, and probably a number of other serovar strains (Table S3) takes place on the outside of the cell and proceeds via the production of a C_55_-P–sugar intermediate that is transported across the membrane and subsequently utilized for the glycosylation of WTA on the outside of the cell (Fig. 10*D*). WTA in *L. monocytogenes* 10403S is decorated with GlcNAc residues, and several *L. monocytogenes* proteins required for the glycosylation of WTA have been identified in previous studies (41, 59–61). One of these is Lmo2550, a GT-A fold glycosyltransferase with a predicted cytoplasmic catalytic domain that is linked via two C-terminal transmembrane helices to the membrane. Lmo2550 shares a high degree of similarity to the *B. subtilis* CsbB protein (*e*-value: 4*e*−91) (59, 60, 62) (Table S3). Hence, it is likely that both proteins, *L. monocytogenes* Lmo2550 and *B. subtilis* CsbB, catalyze the same reaction, which we predict is the formation of a C_55_-P–GlcNAc lipid intermediate in the cytoplasm of the cell (Fig. 10). Another protein required for the WTA glycosylation process in *L. monocytogenes* is Lmo2549 (59, 60). Lmo2549, also named GtcA, is a membrane protein with four predicted transmembrane helices and a GtrA domain. The exact function of GtcA is not known; however, based on homology to small multidrug resistance transporters, GtcA and other members of this family could be involved in the flipping of lipid-linked intermediates across the membrane, as suggested previously (Fig. 10*D*) (63–65). In the case of *L. monocytogenes* 10403S, we hypothesize that this would be the C_55_-P–GlcNAc lipid intermediate. However, it should be noted that biochemical evidence for such flippase activity is still lacking. Finally, the Lmo1079 protein has been implicated in the WTA glycosylation pathway in *L. monocytogenes* (41, 61). Lmo1079 shows a high degree of similarity to the *B. subtilis* transmembrane protein YfhO (*e*-value: 1*e*−39), which, we suggest, is a GT-C-fold glycosyltransferase responsible for the transfer of the GlcNAc residues to the *B. subtilis* LTA polymer on the outside of the cell (Fig. 10*A*). As part of this work, we purified WTA and LTA from an *L. monocytogenes lmo1079* mutant strain and show that this protein is indeed required for WTA glycosylation but not required for the glycosylation of LTA (Fig. 6). It is tempting to speculate that both proteins, *L. monocytogenes* Lmo1079 and *B. subtilis* YfhO, act as extracellular glycosyltransferases and move GlcNAc residues from C_55_-P lipid intermediates onto the WTA or LTA polymers, respectively, on the outside of the cell. Taken together, this suggests that WTA in Gram-positive bacteria cannot only be glycosylated on the inside of the cell as described until now but also on the outside of the membrane (Fig. 10).

LTA in *L. monocytogenes* strain 10403S is modified with galactose residues (22, 23, 29). Because inactivation of Lmo1079, the only clear homolog to the *B. subtilis* YfhO protein in *L. monocytogenes*, did not abolish the glycosylation of LTA (Fig. 6*D*), a different protein must have been responsible for the transfer of galactose residues onto the LTA polymer. Our work indicates that the transmembrane protein GtlB (Lmo0626) is a good candidate protein to catalyze this reaction, as the sugar modifications on LTA were absent in a *gtlB* mutant (Figs. 7*C* and 10*C*). The *B. subtilis* YfhO and *L. monocytogenes* GtlB proteins have different topologies and share little similarity on an amino acid level (Fig. 9, *A* and *B*). Nevertheless, our results show that both proteins are involved in the LTA glycosylation process, and we propose that both proteins transfer C_55_-P–linked sugar residues onto cell wall polymers on the outside of the cell. GtlB is characterized by eight transmembrane helices and a relatively large extracellular loop of 61 amino acids between the first and second predicted TM helix that may be involved in the sugar transfer (Fig. 9*B*). Furthermore, the GtlB protein in *L. monocytogenes* strain EGD-e, which is 100% identical to the *L. monocytogenes* 10403S protein analyzed in this study, is annotated to contain a PMT-2 domain (GenBank^TM^ number CAC98704.1). PMT domains are found in proteins of the dolichyl-phosphate-mannose protein mannosyltransferase family, which catalyze *O*-linked glycosylation of proteins. Members of the PMT family include the l-Ara4-N lipid A transferase ArnT of *Escherichia coli* and *Salmonella typhimurium*, which is responsible for the glycosylation of lipid A, and the Pmt (Cg1014) protein of *Corynebacterium glutamicum*, which is necessary for the glycosylation of secreted proteins (66, 67). Further bioinformatics analysis revealed that the *L. monocytogenes* GtlB protein shows some similarity to the *Pseudomonas aeruginosa* arabinosyltransferase TfpW, which has been described as a GT-C-fold glycosyltransferase associated with the glycosylation of type IV pili (42). These findings strengthen our hypothesis that the *L. monocytogenes* GtlB protein might act as the second glycosyltransferase responsible for the transfer of galactose residues on the outside of the cell to the LTA polymer (Fig. 10*C*), although biochemical evidence is still lacking. GtlB possesses a modified D*XX*D motif in the first predicted extracellular loop that is a characteristic of GT-C glycosyltransferases. Our alanine substitution analysis indicated that both aspartic acid residues, Asp^35^ and Asp^38^, are crucial for GtlB function and for the glycosylation of LTA. A conserved D*XX*D motif has also been identified in other GT-C-type glycosyltransferases, including the WecA protein from *E. coli*, the α-(1–6)-mannopyranosyltransferases Rv1459c and Rv2174 of *M. tuberculosis*, and MptB of *C. glutamicum* (68–70), further highlighting that the enzymatic site of GtlB is probably on the extracellular site of the membrane.

Glycosyltransferases are widely distributed among bacteria. In particular, GT-A-type glycosyltransferases, such as the *B. subtilis* CsbB and *L. monocytogenes* GtlA proteins characterized as part of this and a previous study (29), are found in a large number of bacteria. The distribution of such glycosyltransferases, which are probably responsible for the production of C_55_-P–sugar intermediates, is presented in Table S3 for the different *L. monocytogenes* serovars as well as a selected number of other well-studied Gram-positive bacteria. YfhO and GtlB homologs were not present in all bacteria listed in Table S3. However, we could still identify around 5150 and 1005 proteins that share >20% identity to YfhO of *B. subtilis* and GtlB of *L. monocytogenes*, respectively. YfhO-like proteins are not only found in *Bacillus* and *Listeria* sp., but are also present in a range of other Gram-positive bacteria, including different *Staphylococcus* sp. and *Streptococcus pneumoniae* (Table S3). We would expect that these GT-C-fold enzymes are also involved in the glycosylation of an extracellular cell wall component in these species. However, as highlighted by the findings presented in this study, the exact nature of the cell wall polymer that is modified cannot easily be predicted solely based on homology and will await future experimental studies. GtlB-like proteins are less widely distributed and primarily found in *Listeria* and *Bacillus* sp. Interestingly, the *L. monocytogenes* GtlB protein shares 49% identity with the *B. subtilis* YkoS protein (Table S3). *ykoS* is co-transcribed with *ykoT*, which codes for a GT-A-fold glycosyltransferase that we analyzed as part of this study (Fig. 1*A* and Fig. S1). The *ykoST* operon of *B. subtilis* is expressed during sporulation and regulated by SigG and SpoVT (71) and has also been shown to be under the expression control of the YkoH-YkoG two-component system (72). Neither of these two proteins is required for LTA glycosylation (Figs. S1 and S3) under standard growth conditions, and hence the target for the predicted glycosyltransferases YkoS and YkoT is unknown. However, it is tempting to speculate that the proteins are required for the glycosylation of an extracellular cell wall polymer or perhaps even protein during the sporulation process.

Taken together, we identified several new enzymes required for the glycosylation of LTA in *B. subtilis* and *L. monocytogenes* and speculate that these enzymes function as cytoplasmic or extracellular glycosyltransferases, although biochemical evidence is still lacking. GT-C-fold glycosyltransferases are notoriously difficult to predict bioinformatically, and based on the genetic evidence presented in this work, we suggest that YfhO- and GtlB-like proteins are novel classes of GT-C-fold glycosyltransferases. Taken together, our findings will help us to determine the function of sugar modifications on LTA in Gram-positive bacteria, which has remained elusive up to date. But perhaps most importantly, another implication of our work is that WTA in Gram-positive bacteria might be glycosylated via two different mechanisms, one taking place inside the cell and a second novel mechanism taking place on the outside of the cell.

## Experimental procedures

### Bacterial strains and growth conditions

All strains and plasmids used in this study are listed in Table S1. *E. coli* and *B. subtilis* strains were grown in Lysogeny Broth (LB) medium and *L. monocytogenes* strains in brain heart infusion (BHI) medium at 37 °C unless otherwise stated. If necessary, antibiotics and supplements were added to the medium at the following concentrations: for *E. coli* cultures, ampicillin at 100 μg/ml, chloramphenicol (Cam) at 20 μg/ml, erythromycin (Erm) at 10 μg/ml, and kanamycin (Kan) at 30 μg/ml; for *B. subtilis* cultures, Cam at 5 μg/ml, Erm at 5 μg/ml, and Kan at 10 μg/ml; and for *L. monocytogenes* cultures, Cam at 10 μg/ml, Kan at 30 μg/ml, and IPTG at 1 mm. In this study, we used *L. monocytogenes* strain 10403S and derivatives thereof. However, *L. monocytogenes* EGD-e gene and locus tag numbers were used, as this was the first fully sequenced *L. monocytogenes* strain, and the corresponding 10403S locus tag numbers are given in parenthesis.

### Strain and plasmid construction

All primers used in this study are listed in Table S2. For the construction of *B. subtilis* strains with gene deletions, 1-kb DNA fragments upstream and downstream of the respective gene were amplified, and the products were digested with ApaI and XhoI and ligated to an antibiotic resistance cassette. The antibiotic resistance cassettes were obtained by digesting plasmids pCN34 (Kan), pCN38 (Cam), and pCN49 (Erm) with restriction enzymes ApaI and XhoI. Up- and downstream *ykcC* fragments were generated by PCR using primers ANG1534/1535 and ANG1536/1537, and *ykoS* fragments were generated using primers ANG1564/1565 and ANG1566/1567. The fragments were digested with ApaI and XhoI and ligated with the Cam cassette. Up- and downstream fragments for *ykcB* were generated by PCR using primer pairs ANG1540/1541 and ANG1542/1543, and *csbB* fragments were generated with primer pairs ANG1546/1547 and ANG1548/1549, and these fragments were digested and ligated with a Kan cassette. The up- and downstream regions of *yfhO* and *ykoT* were amplified using primer pairs ANG1552/1553 and ANG1554/1555 and primer pairs ANG1558/1559 and ANG1560/1561, respectively, digested, and fused to an Erm cassette. Subsequently, the purified ligation products were transformed into *B. subtilis* 168, and transformants were selected on LB agar plates containing the appropriate antibiotics. The replacement of the target gene with the antibiotic marker was verified by PCR, resulting in the construction of the *B. subtilis* strains 168Δ*ykcC::cam* (ANG2747), 168Δ*ykcB::kan* (ANG2748), 168Δ*csbB::kan* (ANG2749), 168Δ*yfhO::erm* (ANG2750), 168Δ*ykoT::erm* (ANG2751), and 168Δ*ykoS::cam* (ANG2752). For complementation analysis of the *csbB* mutant, plasmid pDG1662-*csbB* was constructed. To this end, the *csbB* gene, including the upstream promotor region, was amplified from *B. subtilis* 168 chromosomal DNA using primers ANG1624/1625, and the product was digested with BamHI and HindIII and inserted into plasmid pDG1662, which had been cut with the same enzymes. The resulting plasmid pDG1662*-csbB* was recovered in *E. coli* XL1-Blue, yielding strain ANG2905. The *yfhO* mutation in strain 168Δ*yfhO::erm* was complemented by introducing the complete *csbB-yfhO* operon, including the native promotor preceding *csbB*. The *csbB-yfhO* operon was amplified with primers ANG1624/1628, and the fragment was cut with BamHI and HindIII and inserted into plasmid pD1662, yielding plasmid pDG1662-*csbB-yfhO.* In addition, plasmid pDG1662-*csbB_D97A_-yfhO* was produced for the expression of an inactive CsbB variation with an D97A amino acid substitution. For this purpose, PCR products were generated with primer pairs ANG1624/1731 and ANG1628/1732 introducing the required base change in *csbB* as part of the primer sequences. The products were fused by a second PCR using primers ANG1624 and ANG1628. The fragment was digested with BamHI and HindIII and ligated with pDG1662, yielding plasmid pDG1662-*csbB_D97A_-yfhO.* Plasmids pDG1662-*csbB-yfhO* and pDG1662-*csbB_D97A_-yfhO* were recovered in *E. coli* XL1-Blue, yielding strains ANG3508 and ANG3303, respectively. All three complementation plasmids were linearized with XhoI and transformed into *B. subtilis* 168 mutant strains, where the plasmids integrate by double crossover recombination into the *amyE* locus. This resulted in the generation of the *B. subtilis* strains 168Δ*csbB::kan amyE::csbB* (ANG3017), 168Δ*csbB:: kan amyE::csbB_D97A_-yfhO* (ANG3305), 168Δ*yfhO:: erm amyE::csbB-yfhO* (ANG3510), and 168Δ*yfhO:: erm amyE::csbB_D97A_-yfhO* (ANG3304).

For the markerless in-frame deletion of the *L. monocytogenes* genes *lmo1079* (*lmrg*_*00541*) and *lmo0626* (*lmrg*_*00309*, *gtlB*), 1-kb DNA fragments up- and downstream of the respective gene were amplified by PCR using primer pairs ANG1527/1528 and ANG1529/1530 (*lmo1079*) or ANG2516/2517 and ANG2518/2519 (*gtlB*). The resulting PCR products were fused by PCR using the outside primer ANG1527/ANG1530 (*lmo1079*) or ANG2516/ANG2519 (*gtlB*). The products were cut with BamHI and KpnI and ligated with plasmid pKSV7 that had been digested with the same enzymes. This resulted in the generation of plasmids pKSV7-Δ*lmo1079* and pKSV7-Δ*gtlB*, which were recovered in *E. coli* XL1-Blue, yielding strains ANG2793 and ANG4232, respectively. The plasmids were subsequently electroporated into *L. monocytogenes* 10403S, and the genes were deleted by allelic exchange using a procedure described previously (73). The deletion of the respective gene was verified by PCR, resulting in the construction of *L. monocytogenes* strains 10403SΔ*lmo1079* (ANG2794) and 10403SΔ*gtlB* (ANG4264). For complementation analysis of the *gtlB* mutant, plasmid pIMK3-*gtlB* was constructed. Plasmid pIMK3 carries the *P_help_* promoter, which allows for IPTG-dependent gene expression. The *gtlB* gene was amplified using primers ANG2708 and ANG2709, and the product was digested with NcoI and SalI and ligated with pIMK3. Plasmid pIMK3-*gtlB* was recovered in *E. coli* XL1-Blue, yielding strain ANG4401. Additionally, point mutations were introduced into the *gtlB* gene for the expression of GtlB variants with D35A, D38A, and S39A amino acid substitutions. For this purpose, primer pairs ANG2708/2791 (D35A), ANG2708/2902 (D38A), and ANG2708/2793 (S39A) were used to amplify the 5′-end of *gtlB* introducing the respective base changes as part of the primer sequence. The 3′-end of *gtlB* was amplified using primers ANG2709/2790 (D35A), ANG2709/2901 (D38A), and ANG2709/2792 (S39A). The appropriate fragments were fused in a second PCR using primers ANG2708 and ANG2709. The PCR products were cut with NcoI and SalI and ligated with pIMK3 that had been cut with the same enzymes. The resulting plasmids were recovered in *E. coli* XL1-Blue, yielding strains XL1-Blue pIMK3-*gtlB_D35A_* (ANG4630), XL1-Blue pIMK3-*gtlB_D38A_* (ANG4769), and XL1-Blue pIMK3-*gtlB_S39A_* (ANG4631). The pIMK3 derivatives as well as the empty pIMK3 plasmid were transformed into *L. monocytogenes* strain 10403SΔ*gtlB* by electroporation, resulting in the construction of strains 10403SΔ*gtlB* pIMK3 (ANG4637), 10403SΔ*gtlB* pIMK3-*gtlB* (ANG4443), 10403SΔ*gtlB* pIMK3-*gtlB_D35A_* (ANG4648), 10403SΔ*gtlB* pIMK3-*gtlB_D38A_* (ANG4775), and 10403SΔ*gtlB* pIMK3-*gtlB_S39A_* (ANG4638).

### Preparation of cell extract and western blot analysis

*B. subtilis* 168 (WT) and the indicted mutant strains were grown for 20–22 h in 5 ml of LB medium at 30 °C. Bacteria from 4 ml of culture were collected by centrifugation for 30 min at 17,000 × *g*, and bacterial pellets were suspended in 2× SDS-PAGE sample buffer to an *A*_600_ = 3. Samples were boiled for 45 min and centrifuged for 5 min, and 10 μl were loaded onto a 15% SDS-polyacrylamide gel. For LTA detection, the humanized monoclonal LTA antibody (gift from Biosynexus Inc., Gaithersburg, MD) and the HRP-conjugated polyclonal rabbit anti-human IgA, IgG, κ, λ antibody (DakoCytomation) were used at 1:5000 and 1:10,000 dilutions, respectively. *L. monocytogenes* 10403S (WT) and the different mutant strains were grown overnight in 5 ml of BHI medium at 37 °C. The expression of *gtlB* (and the different variants) from the *P_help_* promoter was induced by the addition of 1 mm IPTG to the medium. Cell extracts for LTA detection were prepared as described previously (8). LTA was detected using a polyglycerol phosphate–specific antibody (Clone 55 from Hycult Biotechnology) and an HRP-conjugated anti-mouse IgG (Cell Signaling Technologies) at 1:4000 and 1:10,000 dilutions, respectively. western blots were developed by the enhanced chemiluminescence method, and the signal was detected using a ChemiDoc Touch Imager (Bio-Rad). All experiments were performed at least three times, and representative images are shown.

### LTA and WTA isolation

For the isolation of LTA from *B. subtilis*, strains were grown overnight in 3 liters of LB medium, and cells were collected by centrifugation. For the isolation of LTA from *L. monocytogenes*, the strains were grown overnight in 2–3 liters of BHI medium, and when required, the medium was supplemented with 1 mm IPTG, and the bacteria were harvested by centrifugation. LTA was purified and analyzed using 1D ^1^H NMR, as described previously (7, 29). Briefly, LTA was extracted with butanol and purified by hydrophobic interaction chromatography using a 24 × 1.6-cm octyl-Sepharose column. Fractions containing LTA were identified by western blotting using the humanized monoclonal LTA or polyglycerol phosphate–specific antibody. The LTA-containing fractions were pooled and lyophilized. WTA was purified and analyzed by NMR, as described previously (74). Briefly, bacteria from 5-liter cultures of *L. monocytogenes* 10403S and 10403SΔ*lmo1079* grown to mid-log phage (*A*_600_ of 0.6–1) in BHI medium at 37 °C were collected by centrifugation and subsequently washed with 1 m NaCl of pH 5.8. The cells were disrupted using a bead beater, and the cell debris was collected by centrifugation. After washing the cell wall material with 1 m NaCl, pH 5.8, 0.5% SDS, pH 5.8, and water, pH 5.8, the material was suspended in water and incubated for 30 min at 60 °C. The cell wall material was recovered by centrifugation, washed with water, and suspended in 0.15 mm Tris/HCl, pH 7.0, containing 0.2 mg/ml trypsin and incubated at 37 °C for 18 h. The next day, the material was collected by centrifugation; washed with 1 m Tris, pH 7.0, 1 m Tris, pH 7.0, containing 1 m NaCl, 1 m Tris, pH 7.0; and washed three times with water. The cell wall material was then incubated for 18 h at 4 °C with 10% TCA to hydrolyze the WTA from the peptidoglycan. The peptidoglycan was removed by centrifugation, and the WTA was precipitated with 0.1 volume of 3 m sodium acetate, pH 5.2, and 3 volumes of ice-cold 95% ethanol and left overnight at −80 °C. The WTA was recovered by centrifugation, washed with ethanol, and air-dried. As final step, the WTA was suspended in 1 ml of water and lyophilized.

### NMR analysis of cell wall polymers

To analyze the LTA and WTA polymers by ^1^H NMR, 2 mg of LTA or 6 mg of WTA were suspended and lyophilized twice in 500 μl of D_2_O of 99.96% purity. In the final step, the LTA or WTA preparations were suspended in 500 μl of D_2_O of 99.99% purity, and NMR spectra were recorded on a 600-MHz Bruker Advance III spectrometer equipped with a TCl cryoprobe. To ensure accurate integration of the signals, NMR spectra were recorded at 303 K with a total recycling time of 5 s and a ^1^H flip angle of ∼30°. Three independent LTA and WTA extractions were performed for each strain shown here and once for the data presented in the supporting material. The data were analyzed using Topspin version 3.5 software (Bruker Biospin, Ltd.), and the spectra were annotated according to previously published NMR spectra (29, 37–39, 45, 74–78). To determine the LTA chain length and percentage of d-alanine and sugar modifications, the different peaks were integrated, and chain length and percentage modification calculations were performed for the *B. subtilis* and *L. monocytogenes* LTA as described previously (17, 29). Briefly, the area under the peak at 4.3 ppm corresponding to the CH group of d-alanine was used as a reference and set to 1. The integration values for the peaks of the different LTA components (namely GroP, d-alanine, GlcNAc, or Gal and fatty acids) were determined and adjusted by the number of nonexchangeable protons; for the LTA samples isolated from *B. subtilis*, these are 58 protons for the lipid anchor, 5 for GroP, 4 for d-alanine, and 10 for GlcNAc. For the LTA samples isolated from *L. monocytogenes*, the calculations are based on 58 protons for the lipid anchor, 5 for GroP, 4 for d-alanine, and 7 for Gal modifications. Dividing the proton-adjusted value for GroP by the proton-adjusted value for the lipid anchor gives the chain length of the LTA. To determine the percentage of substitution, the proton-adjusted values for d-alanine and GlcNAc (*B. subtilis*) or d-alanine and Gal (*L. monocytogenes*) were divided by the proton-adjusted value for GroP and then multiplied by 100. The average values and S.D. from three biological replicates were determined and plotted.

### Sequence analysis and protein alignments

For the identification of potential homologs of the *L. monocytogenes* GtlA and GtlB proteins and of the *B. subtilis* YfhO protein, the corresponding protein sequences were used in BLASTP searches against the NCBI non-redundant protein sequence database (79). Sequences of proteins with a minimum query coverage of 60% and a minimum sequence identity of 20% to GtlB were downloaded and used for further analysis in Jalview (80). A multiple-sequence alignment of GtlB and its homologs was performed with Clustal Omega, setting the *L. monocytogenes* GtlB protein as a reference. The alignment was subsequently used to generate a sequence logo motif using the web-based application WebLogo (81).

## Author contributions

J.R., M.G.P., and A.G. conceptualization; J.R., M.G.P., and A.G. resources; J.R. and M.G.P. data curation; J.R., M.G.P., and A.G. formal analysis; J.R. and A.G. funding acquisition; J.R., M.G.P., and A.G. investigation; J.R. and A.G. writing-original draft; M.G.P. and A.G. methodology; M.G.P. writing-review and editing; A.G. supervision; A.G. project administration.

## Supplementary Material

Supporting Information

## Footnotes

5 Please note that the JBC is not responsible for the long-term archiving and maintenance of this site or any other third party hosted site.

## Footnotes

4 The abbreviations used are:

WTA: wall teichoic acid
LTA: lipoteichoic acid
GT: glycosyltransferase
IPTG: isopropyl 1-thio-β-d-galactopyranoside
LB: Lysogeny Broth
BHI: brain heart infusion
Cam: chloramphenicol
Erm: erythromycin
Kan: kanamycin
HRP: horseradish peroxidase.
