# Supplementary material for: Discovery of genes required for lipoteichoic acid glycosylation predicts two distinct mechanisms for wall teichoic acid glycosylation
Source: J Biol Chem. 2018 Jan 17;293(9):3293–306. doi: 10.1074/jbc.RA117.001614 (PMC5836110; doi:10.1074/jbc.RA117.001614)
Supplement: Supporting Information [file supp_293_9_3293__index.html]

Discovery of genes required for lipoteichoic acid glycosylation predicts two distinct mechanism for wall teichoic acid glycosylation — Teichoic acid glycosylation in Gram-positive bacteria — Discovery of genes required for lipoteichoic acid glycosylation predicts two distinct mechanisms for wall teichoic acid glycosylation — Teichoic acid glycosylation in Gram-positive bacteria — Supporting Information 

# Discovery of genes required for lipoteichoic acid glycosylation predicts two distinct mechanisms for wall teichoic acid glycosylation

## Supporting Information

- Supporting Information - Supporting information - Figures S1-S3 and Tables S1-S3
