# Supplementary material for: Discovery of genes required for lipoteichoic acid glycosylation predicts two distinct mechanisms for wall teichoic acid glycosylation
Source: J Biol Chem. 2018 Jan 17;293(9):3293–306. doi: 10.1074/jbc.RA117.001614 (PMC5836110; doi:10.1074/jbc.RA117.001614)
Supplement: Supporting Information [file supp_RA117.001614_134884_1_supp_54414_p2gbrs.pdf]

Supporting Information

Discovery of genes required for lipoteichoic acid glycosylation predicts two distinct mechanism for wall teichoic acid glycosylation

Jeanine Rismondo, Matthew G. Percy, Angelika Gründling

List of Material included:

Supplemental Figure S1
Supplemental Figure S2
Supplemental Figure S3
Supplemental Table S1
Supplemental Table S2
Supplemental Table S3

Supplemental Figures

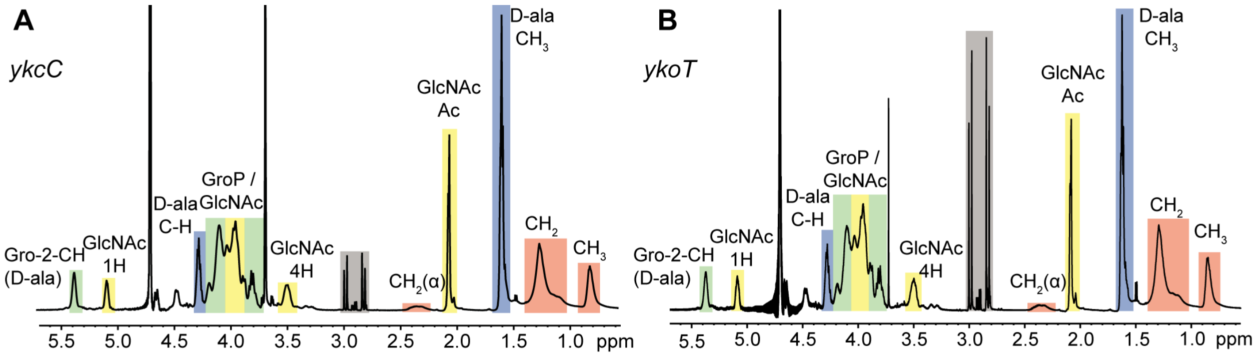

Figure S1. NMR spectra of LTA isolated from *B. subtilis* 168 *ykcC* and *ykoT* deletion strains. (A-B) LTA from the *B. subtilis* 168 (A) *ykcC* mutant and (B) *ykoT* mutant was purified and subsequently analyzed by ^{1}H NMR. Colored boxes and labels indicate nonexchangeable protons of the different LTA components. The different peaks for the protons and acetyl group of GlcNAc are labelled with 1H, 4H and Ac, respectively. Gray boxes indicate protons of citrate, a buffer component used for the LTA purification.

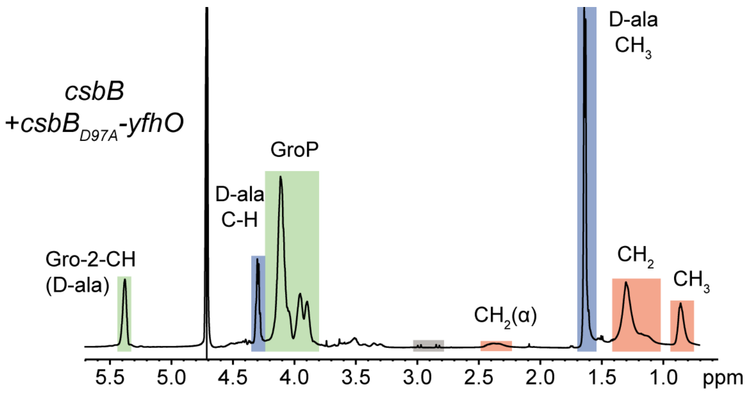

Figure S2. NMR spectrum of LTA isolated from the *B. subtilis* 168 *csbB* mutant strain complemented with the inactive $CsbB_{D97A}$ variant. LTA from *B. subtilis* strain $csbB{+}csbB_{D97A}$-*yfho* was isolated and analyzed by ^{1}H NMR. Colored boxes and labels indicate nonexchangeable protons of the different LTA components. Gray boxes indicate protons of citrate, a buffer component used for the LTA purification.

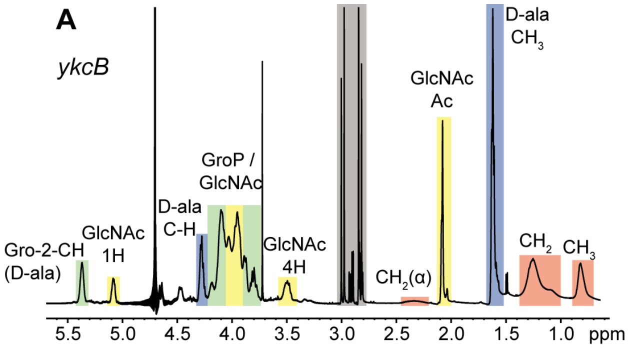

Figure S3. NMR spectra of LTA isolated from *B. subtilis ykcB* and *ykoS* deletion strains. NMR spectra of LTA obtained from the *B. subtilis* (A) *ykcB* mutant and (B) *ykoS* mutant. Colored boxes and labels indicate nonexchangeable protons in the LTA structure. The different peaks for the protons and acetyl group of GlcNAc are labelled with 1H, 4H and Ac, respectively. Gray boxes highlight peaks resulting from residual citrate, a buffer component used for the purification of LTA.

Supplemental Tables

Table S1: Bacterial strains used in this study

Unique ID	Strain name and resistance	Source
***Escherichia coli* strains**		
ANG201	*E. coli* pCN34; AmpR	(3)
ANG203	*E. coli* pCN39; ErmR	(3)
ANG1264	DH5α pKSV7; AmpR	(4)
ANG1676	*E. coli* pCN38; CamR	(3)
ANG2793	XL1-Blue pKSV7-Δ*lmo1079* (*lmrg_00541*)	This study
ANG2802	TG1 pDG1662; AmpR	(5)
ANG2905	XL1-Blue pDG1662-*csbB*; AmpR	This study
ANG3303	XL1-Blue pDG1662-$csbB_{D97A}$-*yfhO*; AmpR	This study
ANG3508	XL1-Blue pDG1662-*csbB-yfhO*; AmpR	This study
ANG4232	XL1-Blue pKSV7-Δ*gtlB* (*lmrg_00309*)	This study
ANG4243	XL1-Blue pIMK3; KanR	(6)
ANG4401	XL1-Blue pIMK3-*gtlB*; KanR	This study
ANG4630	XL1-Blue pIMK3-$gtlB_{D35A}$; KanR	This study
ANG4631	XL1-Blue pIMK3-$gtlB_{S39A}$; KanR	This study
ANG4769	XL1-Blue pIMK3-$gtlB_{D38A}$; KanR	This study
***Bacillus subtilis* strains**		
ANG1691	168, trpC2	(7)
ANG2747	168Δ*ykcC::cam*; CamR	This study
ANG2748	168Δ*ykcB::kan*; KanR	This study
ANG2749	168Δ*csbB::kan*; KanR	This study
ANG2750	168Δ*yfhO::erm*; ErmR	This study
ANG2751	168Δ*ykoT::erm*; ErmR	This study
ANG2752	168Δ*ykoS::cam*; CamR	This study
ANG3017	168Δ*csbB::kan amyE::csbB*; KanR CamR	This study
ANG3304	168Δ*yfhO::erm amyE::*$csbB_{D97A}$-*yfhO*; ErmR CamR	This study
ANG3305	168Δ*csbB::kan amyE::*$csbB_{D97A}$-*yfhO;* KanR CamR	This study
ANG3510	168Δ*yfhO::erm amyE::csbB-yfhO*; ErmR CamR	This study
***Listeria monocytogenes* strains**		
ANG1263	10403S; StrepR	(8)
ANG1386	10403SΔ*ltaS*; StrepR	(9)
ANG2325	10403SΔ*gtlA*; StrepR	(10)
ANG2794	10403SΔ*lmo1079*; StrepR	This study
ANG4264	10403SΔ*gtlB*; StrepR	This study
ANG4443	10403SΔ*gtlB* pIMK3-*gtlB*; StrepR KanR	This study
ANG4637	10403SΔ*gtlB* pIMK3; StrepR KanR	This study
ANG4638	10403SΔ*gtlB* pIMK3-$gtlB_{S39A}$; StrepR KanR	This study
ANG4648	10403SΔ*gtlB* pIMK3-$gtlB_{D35A}$; StrepR KanR	This study
ANG4775	10403SΔ*gtlB* pIMK3-$gtlB_{D38A}$; StrepR KanR	This study

Table S2: Primers used in this study

Number	Name	Sequence
ANG1527	5-BamHI-1kb-*lmo1079*	CGCGGATCCCAAAACGTTCTCCTTCAAAATCATAGGCG
ANG1528	front-int-*lmo1079*	ACGCTTTCTAAGCTTTTTCCATTTTTTTCTAACATCTTCCAT
ANG1529	back-int-*lmo1079*	AAATGGAAAAAGCTTAGAAAGCGTAAAATAAAACACAATAA
ANG1530	3-KpnI-1kb-*lmo1079*	CGGGGTACCGCCCTAAAATCCCAAGAACTCGTGAGCG
ANG1534	5-1kb-YkcC	GTCGCTCTTGTTCATCTGTACCGCAATCAG
ANG1535	front-ApaI-YkcC	CCGGGGCCCCACAGGTACAACAATTGAATATTGAATATG
ANG1536	back-XhoI-YkcC	CCGCTCGAGAAAAGACTCTATAGAGACCAGCATATGTCA
ANG1537	3-1kb-YkcC	CAGCATGCTTGATCTAGAGCAAGTGCCGC
ANG1540	5-1kb-YkcB	GCAGCGACTTGTGCTGACTGCTGATGAGG
ANG1541	front-ApaI-YkcB	CCGGGGCCCGATATCCAGCTCGCGTTTTTCTTTTCCAC
ANG1542	back-XhoI-YkcB	CCGCTCGAGCGGAATGAATCAGTCAGCAACATTATACG
ANG1543	3-1kb-YkcB	GCTGGATCACAATAAGTGATGACCAGCC
ANG1546	5-1kb-CsbB	CATCGGGCTTATTACGCAATTCACTGATG
ANG1547	front-ApaI-CsbB	CCGGGGCCCGACGGGATAATAATCGAGATTAATCCTTGC
ANG1548	back-XhoI-CsbB	CCGCTCGAGGAACGAATTAAAAAGCATGCGGCGTCTGAC
ANG1549	3-1kb-CsbB	CCGGCACAAACGCCGCCGCACTGATGCC
ANG1552	5-1kb-YfhO	GAAGAAGGGTATGACCAAGTCATTGCCC
ANG1553	front-ApaI-YfhO	CCGGGGCCCGTCAAATAATAATTTTTAAATACGATCAT
ANG1554	back-XhoI-YfhO	CCGCTCGAGGCTGTTTTTATATAAGACGTAAAAAGCCC
ANG1555	3-1kb-YfhO	GGCTCCGGATATTGATACTGTGTTAAAGC
ANG1558	5-1kb-YkoT	CTGCTGTTCAAACGAAATCGGACAAAGCC
ANG1559	front-ApaI-YkoT	CCGGGGCCCGACGACGATGGTTAATACTGGCTGTGACTG
ANG1560	back-XhoI-YkoT	CCGCTCGAGCGCCAGGAAAAAGAGCGGTTAGAAAAAAAG
ANG1561	3-1kb-YkoT	TCAGTATATGATTGAAGTCAGTGACTACC
ANG1564	5-1kb-YkoS	GCCCGCCGTGAAAATGCCCTGACAGCAGG
ANG1565	front-ApaI-YkoS	CCGGGGCCCGTTTCTTTTTTTTGTTGTCTCATCAATCGTAAC
ANG1566	back-XhoI-YkoS	CCGCTCGAGGCCTGGCGCATTTATTTATATGAAACAAGATAAG G
ANG1567	3-1kb-YkoS	CAAATGGATATGATAAGAGACGCCCAGCC
ANG1624	5-BamHI-CsbB-with P	CGCGGATCCGCTAAAATTTCTCTCCATCCGTCTGTC
ANG1625	3-HinDIII-CsbB	CCCAAGCTTTCAGTGCATTTTTGTCAGACGCCGCATGC
ANG1628	3-HinDIII-YfhO	CCCAAGCTTCTATATAGAGCCGGGCTTTTTACGTCTT
ANG1731	CsbB-D97A-Fwd	CGGTTATTGTCATGGATGCCGCCCTGCAGCATCCGACATATTTG C
ANG1732	CsbB-D97A-Back	GCAAATATGTCGGATGCTGCAGGGCGGCATCCATGACAATAAC CG
ANG2516	Lmo0626 up fw	GCGCGGATCCCATTATCTGTATCGGGAGTTGG
ANG2517	Lmo0626 up rev	TAAATAGATTTTAATAATTAGCCACTTATGCTTTTTCCACAT
ANG2518	Lmo0626 down fw	TGGCTAATTATTAAAATCTATTTATACAAAGCAACTAATTAA
ANG2519	Lmo0626 down rev	GCGCGGTACCCCTGAGATGATACTGTAGAGATATTC
ANG2708	pIMK3-lmo0626 fw	GCGCCCATGGGGTGGAAAAAGCATAAGTGGCTAATT

ANG2709	pIMK3-lmo0626 rev	GCGCGTCGACTTAATTAGTTGCTTTGTATAAATAGATT
ANG2790	Lmo0626 D35A fw	GATTCATGCTAACTTGGATTCTAATGCGAC
ANG2791	Lmo0626 D35A rev	CCAAGTTAGCATGAATCCTTACATGACTAT
ANG2792	Lmo0626 S39A fw	CTTGGATGCTAATGCGACTTGGTACAAAATG
ANG2793	Lmo0626 S39A rev	GCATTAGCATCCAAGTTATCATGAATCC
ANG2901	Lmo0626 D38A fw	GATAACTTGGCTTCTAATGCGACTTGGTACAAAATG
ANG2902	Lmo0626 D38A rev	GCATTAGAAGCCAAGTTATCATGAATCCTTAC

Table S3: Homologs of *L. monocytogenes* 10403S proteins required for WTA and LTA glycosylation in a select number of Gram-positive bacteria.

L. monocytogenes WTA	Lmo2550 (Lm)	Lmo1079 (YfhO) (Lm)
B. subtilis 168	BSU12890 (YkcC) (2e-95 45%)<br>BSU08600 (CsbB) (2e-86 44%)<br>BSU13390 (YkoT) (4e-50 35%)	BSU08610 (YfhO) (2e-38 23%)
B. cereus ATCC 14579	BC5432 (8e-87 43%)	BC5431 (8e-46 24%)
B. cereus AH820	BCAH820_5528 (2e-88 40%)	-
B. anthracis Ames	BA_5684 (2e-88 40%)	-
B. anthracis Sterne 34F2	BAS5287 (2e-88 40%)	-
L. monocytogenes 10403S (1/2a)	**Lmrg_01697 (0.0 100%)**<br>Lmrg_02032 (6e-87 42%)	**Lmrg_00541 (0.0 100%)**
L. monocytogenes EGDe (1/2a)	**Lmo2550 (0.0 100%)**<br>Lmo0933 (GtlA) (6e-87 42%)	**Lmo1079 (0.0 99%)**
L. monocytogenes SLCC 2755 (1/2b)	**LMOSLCC2755_2556 (0.0 100%)**<br>LMOSLCC2755_0934 (2e-86 42%)	**LMOSLCC2755_1077 (0.0 99%)**
L. monocytogenes SLCC 2372 (1/2c)	**LMOSLCC2372_2614 (0.0 100%)**<br>LMOSLCC2372_0946 (6e-87 42%)	**LMOSLC2372_1093 (0.0 99%)**
L. monocytogenes SLCC 7179 (3a)	**LMOSLCC7179_2462 (0.0 100%)**<br>LMOSLCC7179_0913 (6e-87 42%)	**LMOSLCC7179_1060 (0.0 99%)**
L. monocytogenes SLCC 2540 (3b)	**LMOSLCC2540_2583 (0.0 100%)**<br>LMOSLCC2540_0932 (2e-86 42%)	**LMOSLCC2540_1075 (0.0 99%)**
L. monocytogenes SLCC 2479 (3c)	**LMOSLCC2479_2613 (0.0 100%)**<br>LMOSLCC2479_0945 (6e-87 42%)	**LMOSLCC2479_1092 (0.0 99%)**
L. monocytogenes HCC 23 (4a)	LMHCC_1552 (9e-64 36%)	-
L. monocytogenes F2365 (4b)	LMOf2365_1095 (1e-57 34%)	-
L. monocytogenes SLCC 2376 (4c)	LMOSLCC2376_1047 (8e-64 36%)	-
L. monocytogenes ATCC 19117(4d)	LMOATCC19117_1097 (2e-57 35%)	-
L. monocytogenes SLCC 2378 (4e)	LMOSLCC2378_1092 (1e-57 34%)	-
L. monocytogenes SLCC 2482 (7)	**LMOSLCC2482_2554 (0.0 99%)**<br>LMOSLCC2482_0978 (2e-86 42%)	**LMOSLCC2482_1122 (0.0 99%)**
L. innocua CLIP11262	**Lin2695 (0.0 92%)**<br>Lin1066 (1e-62 33%)	-
L. ivanovii WSLC3009	AX25_04770 (4e-90 43%)	-
L. seeligeri SLCC3954	**Lse_2456 (0.0 95%)**<br>Lse_0831 (2e-89 42%)	**Lse_0969 (0.0 88%)**
S. aureus USA300 FRP3757	SAUSA300_0689 (1e-81 40%)	SAUSA300_1135 (8e-20 22%)
S. epidermidis RP62A	SERP0363 (2e-79 43%)	SERP0809 (1e-25 23%)
E. faecalis ATCC 29212	**DR75_1002 (8e-145 63%)**<br>DR75_1603 (7e-82 38%)	-
E. faecium DO	HMPREF0351_10563 (2e-79 38%)	-
E. faecium T110	M395_02690 (8e-76 38%)	M395_01445 (9e-67 26%)
S. agalactiae NEM316	Gbs1545 (1e-81 38%)	-
S. pyogenes HSC5	L897_02290 (4e-75 36%)	-
S. pneumoniae R6	Spr1459 (GtrB) (3e-80 38%)	Spr2036 (7e-13 29%)
S. pneumoniae Tigr4	SP_1606 (2e-80 38%)	SP_2231 (7e-13 29%)
L. lactis MG1363	Llmg_0134 (2e-80 39%)<br>Llmg_0114 (5e-75 40%)<br>Llmg_0600 (2e-74 41%)<br>Llmg_1521 (3e-62 35%)	-
L. casei BL23	LCABL_12710 (RfaG) (8e-83 38%)<br>LCABL_07730 (YkcC) (6e-81 38%)<br>LCABL_28680 (8e-65 36%)	-
M. tuberculosis H37Rv	Rv1500 (2e-34 28%)	-

Table S3: continued

L. monocytogenes LTA	GtlA (Lm)	GtlB (Lm)
B. subtilis 168	**BSU12890 (YkcC) (3e-158 65%)**<br>BSU08600 (CsbB) (7e-68 39%)<br>BSU13390 (YkoT) (1e-60 41%)	BSU13380 (YkoS) (0.0 49%)
B. cereus ATCC 14579	BC5432 (1e-73 41%)	-
B. cereus AH820	BCAH820_5528 (1e-112 51%)	BCAH820_2601 (0.0 49%)
B. anthracis Ames	BA_5684 (9e-113 51%)	-
B. anthracis Sterne 34F2	BAS5287 (9e-113 51%)	-
L. monocytogenes 10403S (1/2a)	**Lmrg_02032 (0.0 100%)**<br>Lmrg_0.1697 (6e-87 42%)	**Lmrg_00309 (0.0 100%)**
L. monocytogenes EGDe (1/2a)	**Lmo0933 (GtlA) (0.0 99%)**<br>Lmo2550 (6e-87 42%)	**Lmo0626 (0.0 99%)**
L. monocytogenes SLCC 2755 (1/2b)	**LMOSLCC2755_0934 (0.0 99%)**<br>LMOSLCC2755_2556 (6e-87 42%)	**LMOSLCC2755_0632 (0.0 96%)**
L. monocytogenes SLCC 2372 (1/2c)	**LMOSLCC2372_0946 (0.0 100%)**<br>LMOSLCC2372_2614 (6e-87 42%)	**LMOSLCC2372_0635 (0.0 99%)**
L. monocytogenes SLCC 7179 (3a)	**LMOSLCC7179_0913 (0.0 100%)**<br>LMOSLCC7179_2462 (6e-87 42%)	**LMOSLCC7179_0602 (0.0 99%)**
L. monocytogenes SLCC 2540 (3b)	**LMOSLCC2540_0932 (0.0 99%)**<br>LMOSLCC2540_2583 (6e-87 42%)	**LMOSLCC2540_0631 (0.0 96%)**
L. monocytogenes SLCC 2479 (3c)	**LMOSLCC2479_0945 (0.0 99%)**<br>LMOSLCC2479_2613 (6e-87 42%)	**LMOSLCC2479_0631 (0.0 99%)**
L. monocytogenes HCC 23 (4a)	LMHCC_1552 (4e-76 42%)	**LMHCC_2004 (0.0 96%)**
L. monocytogenes F2365 (4b)	LMOf2365_1095 (3e-77 43%)	**LMOf2365_0655 (0.0 96%)**
L. monocytogenes SLCC 2376 (4c)	LMOSLCC2376_1047 (4e-76 42%)	**LMOSLCC2376_0605 (0.0 96%)**
L. monocytogenes ATCC 19117(4d)	LMOATCC19117_1097 (8e-75 43%)	**LMOATCC19117_0656 (0.0 96%)**
L. monocytogenes SLCC 2378 (4e)	LMOSLCC2378_1092 (2e-77 43%)	**LMOSLCC2378_0650 (0.0 96%)**
L. monocytogenes SLCC 2482 (7)	**LMOSLCC2482_0978 (0.0 99%)**<br>LMOSLCC2482_2554 (2e-85 42%)	**LMOSLCC2482_0628 (0.0 96%)**
L.innocua CLIP11262	Lin2695 (3e-87 42%)<br>Lin1066 (9e-80 41%)	**Lin0635 (0.0 93%)**
L. ivanovii WSLC3009	**AX25_04770 (0.0 91%)**	**AX25_03160 (0.0 88%)**
L. seeligeri SLCC3954	**Lse_0831 (0.0 92%)**<br>Lse_2456 (1e-87 43%)	**Lse_0535 (0.0 87%)**
S. aureus USA300 FRP3757	SAUSA300_0689 (3e-70 39%)	-
S. epidermidis RP62A	SERP0363 (5e-69 40%)	-
E. faecalis ATCC 29212	DR75_1603 (1e-87 42%)<br>DR75_1002 (8e-67 36%)	-
E. faecium DO	HMPREF0351_10563 (6e-66 39%)	-
E. faecium T110	M395_02690 (5e-64 40%)	-
S. agalactiae NEM316	Gbs1545 (1e-69 37%)	-
S. pyogenes HSC5	L897_02290 (2e-68 36%)	-
S. pneumoniae R6	Spr1459 (GtrB) (7e-73 38%)	-
S. pneumoniae Tigr4	SP_1606 (1e-72 39%)	-
L. lactis MG1363	Llmg_1521 (4e-72 41%)<br>Llmg_0134 (1e-68 37%)<br>Llmg_0114 (1e-64 38%)<br>Llmg_0600 (5e-64 40%)	-
L. casei BL23	LCABL_07730 (YkcC) (8e-106 49%)<br>LCABL_28680 (2e-68 38%)<br>LCABL_12710 (RfaG) (8e-65 37%)	-
M. tuberculosis H37Rv	Rv1500 (2e-41 30%)	-

L. monocytogenes 10403S proteins used for the BLASTP searches are listed on top of the respective column and query species in the left column. Protein name or locus tag of identified homologs are given with e-value and % identity in parenthesis. Homologs with > 60% identity are shown in bold.

References

1. Waterhouse, A. M., Procter, J. B., Martin, D. M., Clamp, M., and Barton, G. J. (2009) Jalview Version 2--a multiple sequence alignment editor and analysis workbench. *Bioinformatics* **25**, 1189-1191
2. Crooks, G. E., Hon, G., Chandonia, J. M., and Brenner, S. E. (2004) WebLogo: a sequence logo generator. *Genome Res* **14**, 1188-1190
3. Charpentier, E., Anton, A. I., Barry, P., Alfonso, B., Fang, Y., and Novick, R. P. (2004) Novel cassette-based shuttle vector system for gram-positive bacteria. *Appl Environ Microbiol* **70**, 6076-6085
4. Smith, K., and Youngman, P. (1992) Use of a new integrational vector to investigate compartment-specific expression of the *Bacillus subtilis spoIIM* gene. *Biochimie* **74**, 705-711
5. Guerout-Fleury, A. M., Frandsen, N., and Stragier, P. (1996) Plasmids for ectopic integration in *Bacillus subtilis*. *Gene* **180**, 57-61
6. Monk, I. R., Gahan, C. G., and Hill, C. (2008) Tools for functional postgenomic analysis of *Listeria monocytogenes*. *Appl Environ Microbiol* **74**, 3921-3934
7. Burkholder, P. R., and Giles, N. H., Jr. (1947) Induced biochemical mutations in *Bacillus subtilis*. *Am J Bot* **34**, 345-348
8. Bishop, D. K., and Hinrichs, D. J. (1987) Adoptive transfer of immunity to *Listeria monocytogenes*. The influence of in vitro stimulation on lymphocyte subset requirements. *J Immunol* **139**, 2005-2009
9. Webb, A. J., Karatsa-Dodgson, M., and Gründling, A. (2009) Two-enzyme systems for glycolipid and polyglycerolphosphate lipoteichoic acid synthesis in *Listeria monocytogenes*. *Mol Microbiol* **74**, 299-314
10. Percy, M. G., Karinou, E., Webb, A. J., and Gründling, A. (2016) Identification of a Lipoteichoic Acid Glycosyltransferase Enzyme Reveals that GW-Domain-Containing Proteins Can Be Retained in the Cell Wall of *Listeria monocytogenes* in the Absence of Lipoteichoic Acid or Its Modifications. *J Bacteriol* **198**, 2029-2042
